# Chronic Ketamine Administration Modulates Midbrain Dopamine System in Mice

**DOI:** 10.1371/journal.pone.0043947

**Published:** 2012-08-24

**Authors:** Sijie Tan, Wai Ping Lam, Maria S. M. Wai, Wan-Hua Amy Yu, David T. Yew

**Affiliations:** 1 Brain Research Center, Faculty of Medicine, The Chinese University of Hong Kong, Shatin, New Territories, Hong Kong S.A.R., P.R. China; 2 Department of Cell Biology and Anatomy Sciences, The Sophie Davis School of Biomedical Education, City University of New York Medical School, New York, New York, United States of America; Institut National de la Santé et de la Recherche Médicale, France

## Abstract

Ketamine is an anesthetic and a popular abusive drug. As an anesthetic, effects of ketamine on glutamate and GABA transmission have been well documented but little is known about its long-term effects on the dopamine system. In the present study, the effects of ketamine on dopamine were studied *in vitro* and *in vivo*. In pheochromocytoma (PC 12) cells and NGF differentiated-PC 12 cells, ketamine decreased the cell viability while increasing dopamine (DA) concentrations in a dose-related manner. However, ketamine did not affect the expression of genes involved in DA synthesis. In the long-term (3 months) ketamine treated mice, significant increases of DA contents were found in the midbrain. Increased DA concentrations were further supported by up-regulation of tyrosine hydroxylase (TH), the rate limiting enzyme in catecholamine synthesis. Activation of midbrain dopaminergic neurons could be related to ketamine modulated cortical-subcortical glutamate connections. Using western blotting, significant increases in BDNF protein levels were found in the midbrain, suggesting that perhaps BDNF pathways in the cortical-subcortical connections might contribute to the long-term ketamine induced TH upregulation. These data suggest that long-term ketamine abuse caused a delayed and persistent upregulation of subcortical DA systems, which may contribute to the altered mental status in ketamine abusers.

## Introduction

Ketamine has become a popular recreational drug in many parts of the world in recent years due to its psychosis-like effects and cheap prices [1]. In Hong Kong, ketamine abuse has increased rapidly over the last decade and is the most abused psychotropic substance. The most recent data from the Central Registry of Drug Abuse (CRDA) indicated that 31.5% of all the abused drugs are ketamine in Hong Kong in 2011 [2]. Abusive use of ketamine mainly cause mental problems, including anxiety, confusion and memory loss [3], [4]. In the United Kingdom, even unexplained deaths related to ketamine use were reported and the number of cases increased 10 folds from 1999 to 2008 [5].

Pharmacologically, ketamine modulates neurotransmission at postsynaptic receptors such as N-methyl-D-aspartate (NMDA) glutamate receptors and gamma-aminobutryic acid (GABA) receptors. As an uncompetitive antagonist, ketamine blocks NMDA receptor and induces a dissociative anesthesia [6]. Ketamine-induced anesthesia was also thought to work via enhancing the GABA inhibitory neurotransmission in the central nervous system (CNS) [7]. Effects of ketamine on GABA inhibition were further verified by the finding that ketamine potentiated GABA(A) receptors expressed in Xenopus Oocyte at anesthetically relevant or higher concentrations [8]. In our previous study, upregulated GABA(A) receptors were found in prolonged ketamine treated mice brain, suggesting that long-term ketamine administration increased the GABA inhibitory transmission in the CNS [9].

Besides the gluatmate and GABA systems, Kapur and Seeman [10] reported that ketamine exhibited a strong affinity with dopamine (DA) D_2_ receptors, indicating that DA system was also modulated by ketamine. Several studies have demonstrated that a single subanaesthetic dose of ketamine rapidly increased DA release in the prefrontal cortex of rats while repeated ketamine administration increased the basal DA levels, suggesting that there could be a longer duration effects of repeated ketamine on DA concentrations [11],[12]. Since DA neurotransmission was a part of brain reward system, it was a main target of abused drugs [13]. However, little is known about the long-term effects of ketamine on the DA system in the CNS.

In the CNS, DA concentration was largely determined by the DA synthesis rate in dopaminergic neurons. DA was synthesized by hydroxylation of tyrosine to L-3,4-dihydroxyphenylalanine (L-DOPA) by tyrosine hydroxylase (TH) and then L-DOPA was decarboxylated to DA by dopa decarboxylase (DDC). Thereafter, cytosolic DA was transported to synaptic vesicle by vesicular monoamine transporter 2 (VMAT2). Inside the DA vesicles, some of the DA was converted to norepinephrine by dopamine β-hydroxylase (DBH). When the action potential was triggered, catecholamine vesicles fused with cell membrane in the presence of synaptosomal-associated protein 25 (SNAP25) and DA transmission was initiated by release of DA into synaptic cleft [14]. The present study used dopaminergic PC 12 cells and chronic ketamine treated mice to investigate the long-term effects of ketamine on the DA system *in vitro* and *in vivo*. To this end, a fuller elucidation of gene expressions in the enzymes mentioned above is clearly desirable in delineating the molecular changes of the DA system following chronic ketamine use.

## Materials and Methods

### Cell culture and ketamine treatment

Rat pheochromocytoma 12 (PC12) cells were obtained from the American Tissue Culture Collection (ATCC) and were grown in Ham's F-12K (Kaighn's modifications, Invitrogen) medium supplement with 10% heat-inactivated horse serum and 5% fetal bovine serum. Cells were cultured in dishes precoated with 5% poly-l-lysine and incubated in a humidified atmosphere at 37°C and 5% CO2. Culture medium was changed every other day. PC12 cells were treated with NGF (50 ng/ml) and allowed to differentiate for 6 days [15]. The differentiation medium was changed every day. Both undifferentiated and differentiated PC12 cells were exposed to ketamine from 10 to 1000 µg/ml diluted in medium for 24 hours. After treatment, the medium was collected for dopamine determination while the cells were subjected to MTT assays for cell viability or used for RNA extraction.

### MTT assay

MTT assay was performed as previously reported [16]. After cells were treated as above mentioned, 100 µl of culture medium containing 0.5 mg/ml Thiazoyl blue tetra-zolium bromide (MTT) was added and incubating at 37°C for 4 hours. Then the medium was aspirated off carefully without disturbing the crystal and 100 ul DMSO were added to each well. After 15 min incubation, optical density (OD) was determined using an automatic microtiter plate reader (Epson LX-800, Molecular Devices) at wavelength of 550 nm.

### Animals and ketamine administration

All animal experiments were approved by the Animal Experimentation Ethics Committee (AEEC) of the Chinese University of Hong Kong (CUHK) and were performed under license of the Department of Health, the Government of the Hong Kong SAR, according to the Animals (Control of Experiments) Ordinance Chapter 340(Animal License ID: (10–297) in DH/HA&P/8/2/1 Pt.13). ICR mice were housed in the Laboratory Animal Services Center (LASEC) of the CUHK, with temperature at 22°∼24°C and humidity level of 45%∼55% under 12-hour alternating light-dark cycles. Standard diet (PicoLab Rodent Diet 20, PMI Nutrition Inc., Henderson, USA) and water were given ad libitum. Ketamine (Alfasan Inc., Utrecht, Holland) was given through intraperitoneal injection daily at a sub-anesthetic dose of 30 mg/kg while controls received same volume of normal saline. Body weights of the mice were measured weekly for dose adjustment. After 3 months, mice were sacrificed by cervical dislocation and the brain tissues were collected for subsequent experiments.

### Dopamine determination

Dopamine concentrations in the brain tissues and cell culture were evaluated using Dopamine Research ELISA™ (BA E-5300, Nordhorn, Germany) according to manufacturer's instructions. Following ketamine treatment, cell culture medium was measured directly. For brain tissues, 50 mg of tissue were homogenized in 1 ml HCl (0.01 N) with EDTA (1 mM) and sodium metabisulfite (4 mM). Under this condition, DA is positively charged and has the optimized solubility. The homogenate was centrifuged at 15000 g at 4°C for 15 min and the supernatant was collected for measuring. 20 µl of standards and diluted sample were used for measurements. The process included Cis-diol-specific affinity gel extraction, acylation, derivatization enzymatically. Finally, DA was detected by competitive ELISA. Results were obtained from microplate reader set to 450 nm and a reference wavelength between 620 nm and 650 nm. The concentrations of DA in the sample were calculated according to the six standards from 0 to 90 ng. By using the ELISA method, this kit provided very sensitive (lower limits at 0.7 ng/mL) and high throughput measurements of DA.

### Real-time PCR

Quantitative real-time PCR (qRT-PCR) was performed as previously described [9]. Briefly, total RNA was isolated using RNeasy Lipid Tissue Mini Kits (Qiagen Inc., Valencia, CA, USA) according to the manufacturer's manual. First strand cDNA synthesis was performed by using a RT^2^ First Strand Kit from Qiagen in a 20 µl reaction volume. 50 µl of PCR volumes were composed of 25 µl Power SYBR® Green PCR Master Mix kit (Applied Biosystems, Foster City, CA, USA), 1 µl cDNA and 1 ul primers (500 µmol/l). The primers of TH (5′GAG TTT GAC CCT GAC CTG GAC 3′, 5′CTC ACC CTG CTT GTA TTG GAA3′), DDC (5′ CGC AAG TGA ATT CCG AAG GA 3′, 5′ ACC TGG CGT CCC TCA AT 3′), SNAP25(5′ CCA TCT CCC TGT GGT TTG TCA 3′, 5′CAG CAA TTT GGT TGT GCA TAG C 3′), VMAT2(5′ CGA GCA TCT CTT ATC TCA TTG GA 3′, 5′ ATA GCC ACC TTC CCA TTT TGT G 3′), DBH(5′ACC GGC TAC TGC ACA GAC AAG 3′, 5′ TCC TGC CCG TCA GGT GTG T3′) and β-Actin(5′ AGG CCA ACC GTG AAA AGA TG 3′, 5′ ACC AGA GGC ATA CAG GGA CAA 3′) were designed in Primer Premier 5.0 (Premier Biosoft International, Palo Alto, California, USA). The amplifications were performed in 96 well plates in a 7900HT Fast Real-Time PCR System (Applied Biosystems, Foster City, CA, USA). Specificity of amplification was confirmed by the melting curve with one peak in PCR reactions. Gene expression changes between the ketamine and saline groups were determined according to 2^-Delta Delta Ct^ method using β-Actin as endogenous control [17].

### Western blotting

Brain tissues were homogenized in 300 µl RIPA lysis buffer containing protease inhibitor cocktail (Millipore Inc., Billerica, MA, USA). The homogenate was centrifuged at 14,000 g for 30 min at 4°C and the supernatants were collected for following WB experiments. Protein concentration of the extracts was determined by the Bio-Rad DC protein assay (Bio-Rad Laboratories Inc., Hercules, CA, USA). 50 µg of protein were loaded on 10% SDS-PAGE gel. Electrophoresis was performed at 100 Volt (V) for 2 hours and followed by transferring onto PVDF membrane at 200 mA for 60 min. The membrane was blocked with 5% non-fat dry milk for 1 h at room temperature and then incubated overnight at 4°C with f\primary antibodies: anti-BDNF (1∶1,000) (AB1779SP), anti-β-actin (1∶20,000) (MAB1501). Both antibodies were obtained from Millipore (Millipore, Billerica, MA). In the following day, the membrane was washed with 0.05% Tween-20 and phosphate buffered saline three times and then incubated with the corresponding horseradish peroxidase (HRP) conjugated secondary antibody (Dako Corporation) at room temperature for 1 hour. Blots were then developed using ECL Plus Kit (Millipore) on Fuji Medical X-ray film and scanned in a Bio-Rad 6500 scanner. Optical density was obtained with Quantity One software (Bio-Rad).

### Immunohistchemistry

For immunostainning, all samples were fixed in 4% paraformaldehyde. The samples were then dehydrated in graded concentrations of ethanol, cleared in xylene, embedded in paraffinwax and sectioned at 5 µm. To detect TH protein, brain sections were deparaffinized, rehydrated, and endogenous peroxidase were blocked with 10% hydrogen peroxide (H_2_O_2_) in absolute methanol for 30 minutes. Sections were permeabilized (antigen retrieval) with 1x phosphate buffered saline (PBS) supplemented with 0.1% Triton-X and 0.05% Tween 20 for 10 min. After three rinses in 1 x PBS, non-specific binding was suppressed by 1.5% normal blocking serum for 30 min. Thereafter, the sections were incubated at 4°C overnight with rabbit anti-Tyrosine Hydroxylase (AB152, Millipore, Billerica, MA) diluted 1∶2000 in blocking solution. On the next day, sections were washed three times with 1 x PBS (5 min each) and incubated with biotinylated secondary antibody (1∶1000 diluted in blocking solution) for 1 hour at room temperature. Subsequently, sections were washed three times in 1 x PBS (5 min each) again and the sections was incubated with streptavidin-HRP(1∶1000 diluted in PBS) for 1 hours. After three times of rinse with 1 x PBS, the colors were developed by 0.05% 3′3′-diaminobenzidine tetrahydrochloride (DAB) in PBS containing 0.01% H_2_O_2_ and finally the sections were dehydrated and mounted.

### Statistical analysis

Significance of differences between control and treatment groups were compared by student's t-test. Differences were considered significant when p<0.05. For real-time PCR results, the p values were calculated based on the replicates 2?(- Delta Ct) values for each gene in the control group and treatment group. Calculations were done using SPSS software (version 15.0).

## Results

### Dose-related effects of ketamine on dopamine concentrations in PC12 and NGF differentiated PC12 (d-PC12) cells

**Figure 1:**
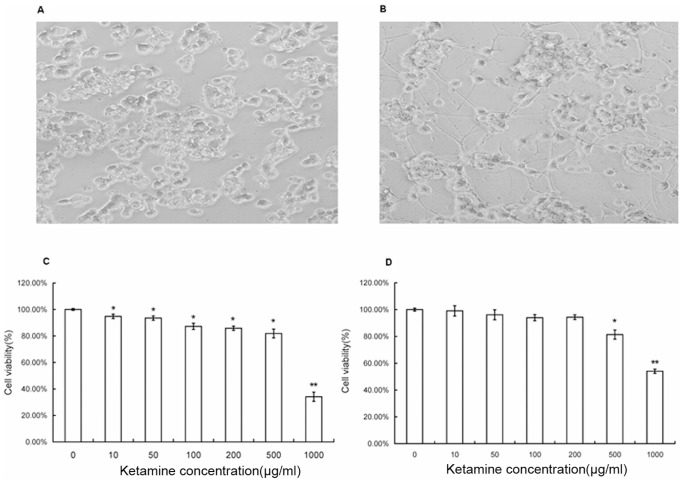
Cell viability of pheochromocytoma 12 (PC12) and differentiated PC12 cells following 24 hours ketamine treatment. A) Phase contrast image of undifferentiated PC12 cells. B) Phase contrast image of PC12 cells differentiated with nerve growth factor (NGF) (50 ng/ml) for 6 days. C) Dosage effects of ketamine on cell viability in undifferentiated PC12 cells. D) Dosage effects of ketamine on cell viability in differentiated PC12 cells. Data are presented as Mean± S.E.M. * *p*<0.05 as compared with control; **p<0.005 as compared with control.

PC 12 cells (Figure 1A) are dopaminergic cells and it can be differentiated into neuronal cells with nerve growth factor (NGF), which make it a useful model for neurobiological and neurochemical studies [18]. After 6 days of NGF treatment, PC12 cells were differentiated into neuronal like cells, with round cell bodies and abundant neurites (Figure 1B). Both the PC12 cells and d-PC12 cells were used for testing the effect of ketamine. After 24 hours treatment, ketamine at 10 µg/ml or higher caused significant decrease in cell viability in PC12 cells (p<0.05 as compared with the control); Ketamine at 1000 µg/ml decreased the cell viability to 34%±3.5% of their control (p<0.005) (Figure 1C). In d-PC12 cells, significant cell viability decreases were found at 500 µg/ml or higher (p<0.05); Ketamine at 1000 µg/ml decreased the cell viability to 54%±1.6%(p<0.005) (Figure 1D). These results indicated that d-PC12 cells were less susceptible to ketamine's toxicity than undifferentiated PC12 cells.

**Figure 2:**
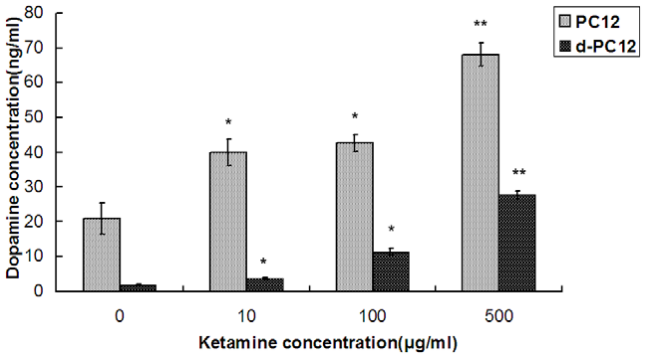
Dosage effects of ketamine on dopamine in pheochromocytoma 12 (PC12) cells and differentiated PC12 cells (d-PC12). PC12 cells and d-PC12 cells were exposed to ketamine from 10 to 500 µg/ml for 24 hours. After the treatment, dopamine concentrations were measured using ELISA. Data were presented as Mean± SEM (n = 3). * *p*<0.05 as compared with control; **p<0.005 as compared with control.

To explore the dosage effects of ketamine on dopamine, extracellular dopamine concentrations following 24 hours exposure to different concentrations of ketamine were measured using ELISA. It was found that ketamine increased dopamine levels in PC12 cells in a dose-related pattern, with dopamine increase from 20.8±2.0 ng/ml (control) to 39.9±3.7 ng/ml (p<0.05) for10 µg/ml ketamine, 42.6±4.4 ng/ml (p<0.05) for 100 µg/ml ketamine and 67.9±5.6 ng/ml (p<0.005) for 500 µg/ml ketamine respectively. Similar dose-related effects were also found in d-PC12 cells (Figure 2). Dopamine concentrations reversely correlated with the cell viability indicated that toxic effects of ketamine to the cells might be related partially to dopamine-induced reactive oxygen species [19].

### Long-term effects of ketamine on dopamine in mouse brain

**Figure 3:**
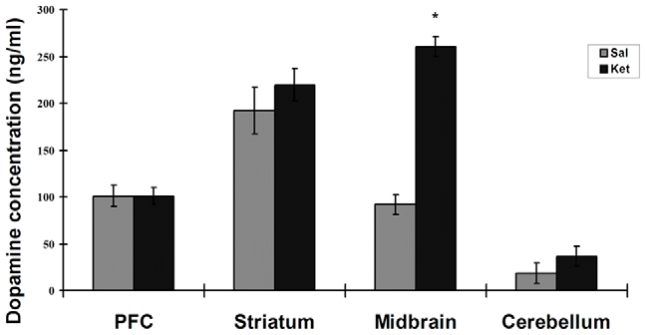
Different dopamine concentrations in mouse brain following 3 months ketamine treatment. Ketamine (30 mg/kg) were given to the mice for 3 months. Dopamine contents were measured by ELISA. Significant dopamine level change was found in the midbrain. PFC, prefrontal cortex. Sal, saline group; Ket, ketamine group; Data were presented as Mean± SEM (n = 6). * *p*<0.05 as compared with control.

To investigate the long-term effects of ketamine on dopamine concentrations, dopamine levels were determined in 4 brain regions (prefrontal cortex, striatum, midbrain and cerebellum) of mice receiving ketamine for 3 months. In the saline (control) group, the highest concentration of dopamine was found in striatum (192.4±25.0 ng/ml) and lowest dopamine in the cerebellum (18.7±10.8 ng/ml) (Figure 3). When compared with the saline group, dopamine levels were found significantly increased in the midbrain of ketamine group (saline, 91.9±10.5 ng/ml; keamine, 260.9±10.5 ng/ml, *p*<0.05). Increased dopamine levels in brain suggested dopaminergic hyperactivity following long-term ketamine treatment.

### Effects of ketamine on mRNA levels of dopamine related genes *in vitro* and *in vivo*

**Figure 4:**
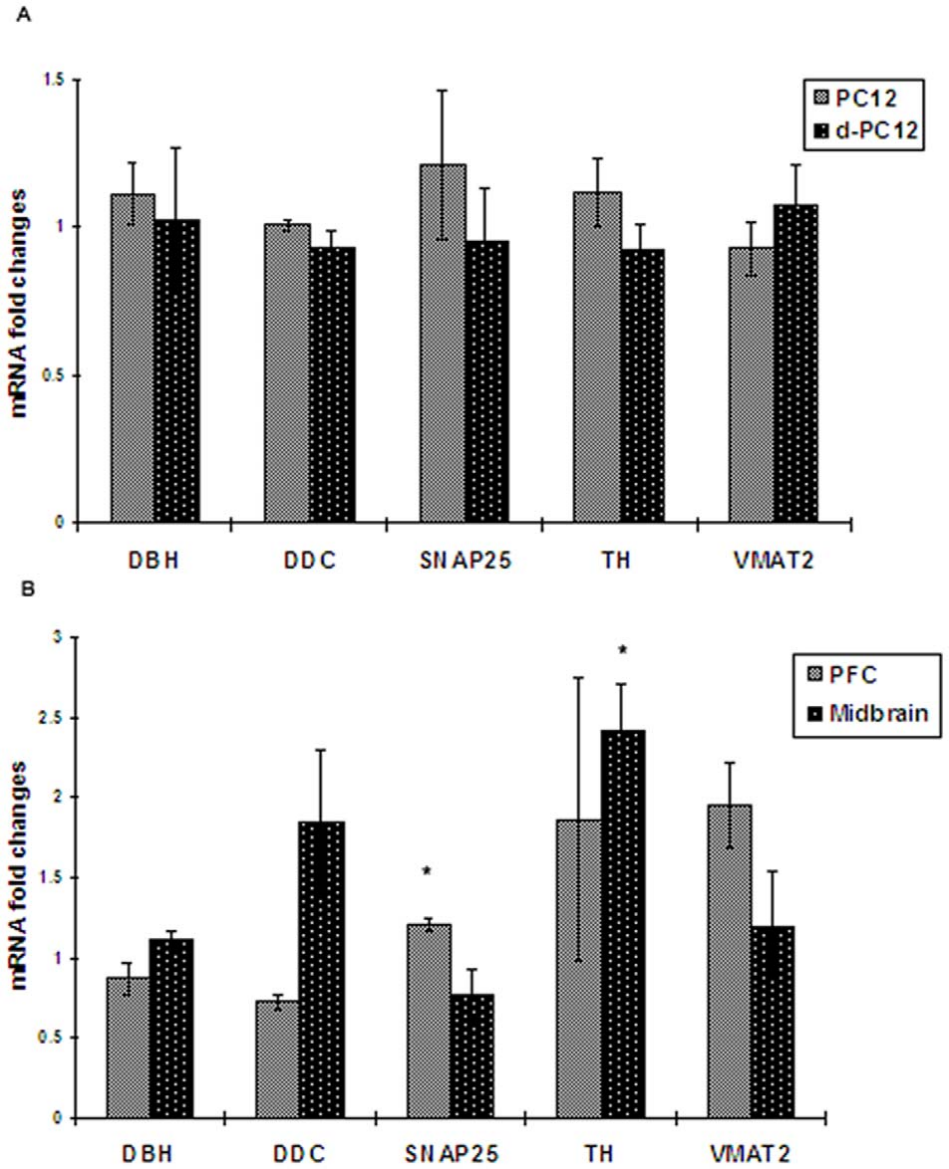
Effects of ketamine on dopamine-related genes *in vitro* and *in vivo*. A) Changes in dopamine-related genes in pheochromocytoma 12 (PC12) and differentiated PC12 (d-PC12) cells. Cells were exposed to ketamine (100 µg/ml) for 24 hours. mRNA levels were measured by real-time PCR and fold changes were calculated using mRNA levels in ketamine group over saline group. B) Changes in dopamine-related genes in mouse brain following 3 months ketamine treatment. DBH, dopamine beta-hydroxylase; DDC, dopa decarboxylase; SNAP25, synaptosomal-associated protein 25; TH, Tyrosine hydroxylase; VMAT2, vesicular monoamine transporter 2; PFC, prefrontal cortex. Significant gene expression changes were found for SNAP25 in PFC and TH in midbrain. Data were presented as Mean± SEM (n = 4). * *p*<0.05 as compared with control.

Expression of dopamine-relate genes (TH, DDC, SNAP25, VMAT2 and DBH) were evaluated in ketamine treated PC12 cells and d-PC12 cell, but no significant change was found (Figure 4A). To further investigate the long-term effects of ketamine on dopamine system, mRNA levels of dopmaine metabolism-related genes were measured by real-time PCR in the PFC and midbrain. In the PFC, measurements of VMAT2 and SNAP25, which were molecules involved in dopamine release, were upregulated to1.9±0.5 folds (p>0.05) and 1.2±0.03 folds (p<0.05) when compared with the control respectively; TH was upregulated 1.8±0.9 folds (p>0.05) of the control. In the midbrain, DA synthesis enzymes TH and DDC were upregulated to 2.4± 0.3 folds (p<0.05) and 1.8±0.4 folds (p>0.05) while VMAT2 and SNAP25 were 1.2±0.3folds (p>0.05) and 0.8±0.2 folds (p>0.05) respectively (Figure 4B).

### Long-term effects of ketamine on BDNF protein levels in mouse brain

**Figure 5:**
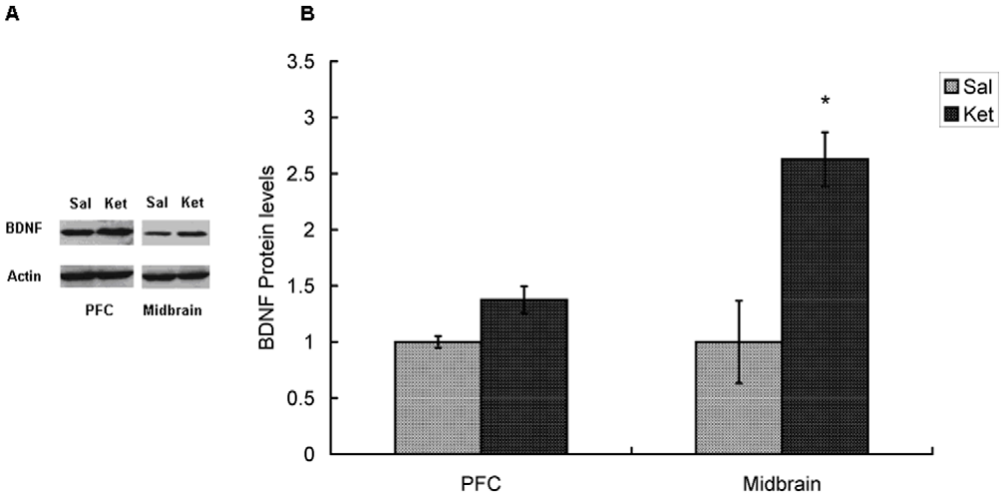
Increase in Brain-derived neurotrophic factor (BDNF) protein expression in mouse brain following 3-month ketamine administration. Sal, saline group; Ket, ketamine group; A) Representative immunoblots of BDNF and Actin. B) Quantification of the increase in BDNF in ketamine treated mice brain. Data are mean ± SEM (n = 6). *P<0.05 as compared with control group.

Brain-derived neurotrophic factor (BDNF) is a widely distributed neurotrophin in the central nervous system. A recent study showed that tyrosine hydroxylase (TH) gene transcription was activated by BDNF through TrkB and the ERK/MAP kinase pathway [20]. To explore the mechanism of ketamine induced TH up-regulation, BDNF protein levels were determined by western blotting in PFC and midbrain. Representative immunoblots and quantification results were shown in Figure 5. It was found that BDNF protein levels were up-regulated in both brain regions (PFC, 1.38±0.12 folds, p = 0.12; midbrain, 2.63±0.24 folds, p = 0.005) after 3 months of ketamine treatment. These results suggested that BDNF related pathway might play a role in the up-regulation of TH in ketamine treated mice.

### Increase of TH inmmureactive neurons in midbrain following 3 months ketamine treatment

**Figure 6:**
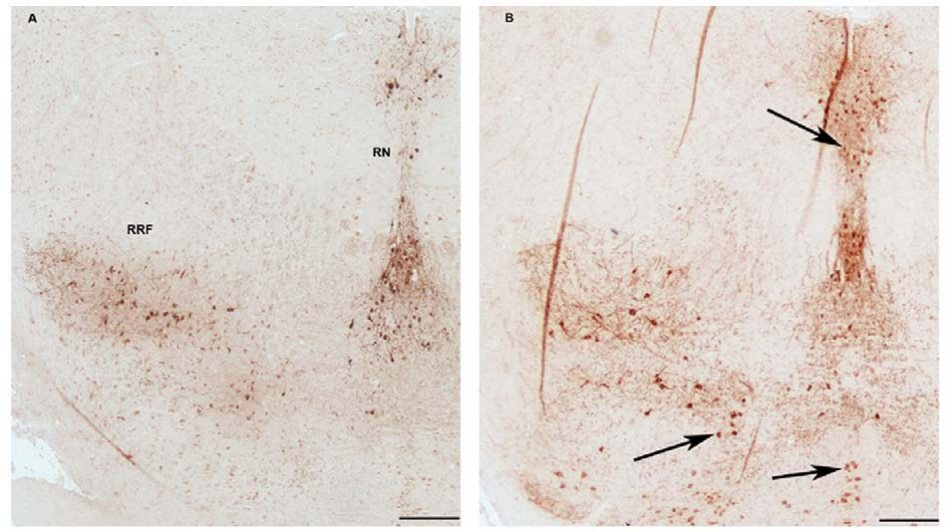
Tyrosine hydroxylase (TH) immunostaining of sections from the midbrain of mice receiving ketamine for 3 months and control mice receiving no ketamine. TH positive dopaminergic neurons were observed in the median ventral tegmental area (VTA) and raphe nucleus (RN). In ketamine treated mouse, increased TH positive neurons were found in RN and other regions (right figure). Scale bars: 200 µm.

In TH immunostaining of sections from the midbrain of mice, TH positive neurons were observed in retrorubral field (RRF) and median raphe nucleus (MRN) (Fig.6 left). The section of midbrain from ketamine treated mouse revealed increased numbers of TH neurons in MRN and RRF (Figure 6A). Moreover, scattered distributions of TH positive neurons were also observed in the regions where TH inmmureactively was not present in the control (Figure 6B).

## Discussion

Ketamine, a glutamate receptor antagonist, is an anesthetic and also a drug of abuse. Recent studies suggested that ketamine affected not only the glutamate and GABA system but also may influence the dopamine system. In the present study, we examined the effects of ketamine on the dopamine system *in vitro* and *in vivo*. Employing dopaminergic PC12 cells, we found that acute exposure to ketamine decreased cell viability and increased DA efflux without altering related gene expressions. The increased total DA levels were correlated with up-regulation of the DA synthesis related enzymes in brain following long-term (3 months) ketamine administration. These results lend support to the hypothesis that long-term ketamine abuse lead to DA dysregulation in the CNS.

In this study, DA concentrations in PC12 cells were found to be reversely related with cell viability after ketamine treatment. As an abusive drug, the toxic effects of ketamine have been well documented *in vitro* [16], [21], [22]. It was reported that ketamine activated mitochondria apoptotic pathway and induced cell apoptosis in cultured cortical neurons through NMDA receptor NR1 subunit [21]. Increased NMDA receptors were known to alter the ion and water homeostasis in the cell and thus caused cell membrane damage [23], which was the hallmark of necrosis. Cell necrosis following ketamine treatment was confirmed by a marked increase of lactate dehydrogenase (LDH) leakage in neuronal cells as well as human hepatoma Hepg2 cells [22], [24].Thus, cell necrosis might also contribute to ketamine's toxic effects. PC12 cells were dopminergic cells and loss of integrity in the plasma membrane would lead to DA leakage without activation of Ca^2+^ dependent DA release. Besides, ketamine has also been reported to promote spontaneous DA release, a process without extracellular Ca^2+^ [25]. Ketamine at 10 ug/ml or higher in concentration caused significant cell death in PC12 cells and corresponding increases of DA. However, ketamine at lower concentration (10 ug/ml and 100 ug/ml) did not cause significant cell death but still increased DA levels in d-PC12 cells, indicating that ketamine promoting spontaneous DA efflux might play a role. These results suggested that ketamine induced DA efflux *in vitro* was caused, at least in part, by its cytotoxicity which lead to damages to plasma membranes.

Although *in vitro* studies showed that ketamine promotes DA release without activation of the dopaminergic transmitter machinery, *in vivo* effects of ketamine on the DA system may be more complex when taking neural circuit system into consideration. Acute effects of ketamine on DA system mainly depend on cortical-subcortical circuit connections. As is well known, most of the DA in CNS is derived from projections of dopaminergic neurons in the ventral tegmental area/substantia nigra (VTA/SN) and raphe nucleus (RN), which are localized in the midbrain [26]. *In vivo*, activation of dopaminergic neurons in midbrain are regulated by cortical glutamatergic projections through a facilitatory pathway and an inhibitory pathway. The facilitatory pathway is mediated by NMDA and AMPA/kainate receptors while the inhibitory pathway is controlled by NMDA receptor via GABA interneurons [27]. As an NMDA receptor antagonist, ketamine not only down-regulates the inhibitory pathway but also activated glutamate neurotransmission at AMPA/kainate receptors through inducing glutamate efflux [12]. Following even a single subanesthetic ketamine injection, midbrain DA neurons were activated by increased net excitatory inputs and rapid DA efflux was evoked in the PFC [11], [12]. Moreover, recent study showed that these cortical-subcortical glutamate connections were indispensable to midbrain DA neuron activation since ketamine did not evoke DA release in cortical slices when this connection was severed [28]. As an abusive drug, ketamine is used repeatedly by addicts and whether there are long-lasting effects of ketamine on the DA system or not need to be elucidated *in vivo*. We measured DA content in brain tissues from long-term ketamine abused mice [9], [29] and noted a significant increase of DA concentration in the midbrain and slightly (but not significantly) increases of DA contents in the striatum and cerebellum after 3 months of ketamine administration (Figure 3).

It is noteworthy that increased DA contents depended on up-regulation of mRNA levels in DA related genes (Figure 4). TH is the rate limiting enzyme in DA biosynthesis and its activity plays an important role in determining dopamine concentrations [30]. We observed that there was significant up-regulation of TH expression (2.4 folds) in the midbrain following a period of 3 months of ketamine treatment. Up-regulation of TH expression was also found in animals acutely or chronically treated with other abusive drugs such as morphine and phencyclidine [31], [32]. However, our results in PC12 cells showed that there was no difference of TH expression following ketamine treatment. Given the difference in the results between *in vitro*/*in vivo* experiments, repeated and intact neuronal circuit inputs modulating the activity of midbrain DA neurons had to be considered to explain the long-term effects of ketamine on TH expression in the animal model. Subanesthetic doses of ketamine activated midbrain DA neurons through evoking glutamate efflux and stimulating AMPA/kainate receptors. Several studies have demonstrated that AMPA/kainate receptor increased BDNF expression via either Ca^2+^ singnaling pathway or mitogen-activated protein kinase (MAPK) pathway [33], [34]. Available evidences indicate that neurotrophic factors including glial cell-derived neurotrophic factor (GDNF) and BDNF increase TH activity and DA synthesis [20], [35]. In western blotting study, we observed significant increases of BDNF protein levels in midbrain, suggesting that BDNF pathways may contribute to long-term ketamine induced TH upregulation.

In summary, besides the well-described transient effects of ketamine on DA release, the present study indicated that long-term ketamine abuse caused a more delayed and persistent upregulation of subcortical DA system. Our data suggested the possibility that upregulation of TH expression represented a common molecular adaptation in the DA system. A better understanding of these ketamine-mediated modifications of DA transmission may lead to the direction of pharmacotherapies for ketamine intoxications, and targeting the DA pathways could be taken into consideration in devising therapeutic approaches for chronic ketamine abusers.
